# nWASP Inhibition Increases Wound Healing via TrKb/PLCγ Signalling

**DOI:** 10.3390/biom13020379

**Published:** 2023-02-17

**Authors:** Bethan A. Frugtniet, Fiona Ruge, Andrew J. Sanders, Sioned Owen, Keith G. Harding, Wen G. Jiang, Tracey A. Martin

**Affiliations:** 1 Division of Cancer and Genetics, Cardiff University School of Medicine, Cardiff University, Cardiff CF14 4XN, UK; 2 Institute of Biomedical Science, University of Gloucestershire, Cheltenham GL50 2RH, UK; 3 School of Applied Sciences, University of South Wales, Pontypridd CF37 4AT, UK; 4 Wound Healing Research Unit, Cardiff University School of Medicine, Cardiff University, Cardiff CF14 4XN, UK

**Keywords:** wound healing, nWASP, therapy, chronic wounds, motility

## Abstract

(1) Background: Chronic wounds represent a major burden to patients and healthcare systems and identifying new therapeutic targets to encourage wound healing is a significant challenge. This study evaluated nWASP as a new therapeutic target in human wound healing and determined how this can be regulated. (2) Methods: Clinical cohorts from patients with chronic wounds were tested for the expression of nWASP and cell models were employed to evaluate the influence of nWASP on cellular functions that are key to the healing process following knockdown and/or the use of nWASP-specific inhibitors. (3) Results: nWASP was significantly elevated at transcript levels in human non-healing chronic wounds versus healing tissues. nWASP inhibitors, wiskostatin and 187-1, along with the knockdown of nWASP, modified both HaCaT and HECV cell behaviour. We then identified two signalling pathways affected by nWASP inhibition: TrkB signalling and downstream PLCγ1 phosphorylation were impaired by nWASP inhibition in HaCaT cells. The healing of wounds in a diabetic murine model was significantly improved with an nWASP inhibitor treatment. (4) Conclusions: This study showed that nWASP activity was related to the non-healing behaviour of chronic wounds and together with the findings in the in vivo models, it strongly suggested nWASP as a therapeutic target in non-healing wounds that are regulated via TrkB and PLCγ1 signalling.

## 1. Introduction

Wound healing can be described as the process of the repair of cutaneous tissue damage or breakdown. It consists of a complex cascade of events that can be summarised into several overlapping stages, each requiring coordination of a variety of signals and cell types [1,2,3,4,5,6]. Wound remodelling involves many cells and signalling pathways and consists of the critical re-epithelialisation of the wound by keratinocytes as they proliferate and migrate in order to close the wound. This is achieved through cell polarisation in the direction of movement according to numerous signals, such as cytokines and growth factors, and the extension of actin-rich membrane structures [7]. Furthermore, adhesion dynamics, where a coordinated balance in the formation of adhesions at the leading edge of the cell and disassembly at the rear, allows cells to gain traction by connecting the actin cytoskeleton to the surrounding matrix, subsequently enabling the cell to move. An optimal level of adhesion is key in allowing the motility of migrating cells [6,8,9,10].

In most cases, acute cutaneous wounds, which typically occur suddenly or following surgery, close within a reasonable period. However, underlying pathological complications or other factors that impair the normal healing process can cause chronic or non-healing wounds to form. Many factors, such as vascular compromise, repetitive insult to the tissue or chronic inflammation, can lead to chronic wound formation. This makes aged individuals or patients with diabetes, vascular diseases and auto-immune diseases more vulnerable to developing chronic wounds, the majority of which are classified as leg, pressure or diabetic foot ulcers [11,12,13,14,15,16]. Chronic non-healing wounds present a substantial economic burden to the healthcare system; significant reductions in quality of life for those affected; and often precede serious events, such as limb amputations or even premature deaths [17,18]. In addition to the significant costs associated with these wounds, they also have a large impact on the quality of life of patients. Physical symptoms from chronic wounds include leakage, odour and pain, as well as impairments to daily living [19,20,21]. The lack of appropriate investigation and classification of chronic wounds was identified by several studies and healthcare professionals currently often rely on limited, traditional wound care approaches, such as dressings, debridement and compression treatment [22,23,24]. There is a clear need for effective diagnosis and identification of new therapeutic approaches to treat chronic wounds. The complex and diverse underlying genetic and molecular processes involved in the wound healing process and chronic wound development make identifying the cause of a particular chronic wound a difficult task. However, these genetic and protein expression deficiencies in chronic wounds also represent potential diagnostic and therapeutic opportunities. Several studies explored these molecular changes and, as a result, highlighted many novel proteins that could potentially be used to explain the development of chronic wounds, offer prognoses and even influence wound repair [25,26,27]. In one study, nWASP, amongst other molecular markers, was shown to have differential expression in acute, chronic healing and chronic non-healing wound tissues [27].

nWASP (neural Wiskott–Aldrich syndrome protein) belongs to the WASP/WAVE (WASP family verprolin-homologous protein) family. Mutations in the first WASP family member to be identified, namely, WASP itself, were found to be the cause of WAS, which is a recessive disorder that was initially described as a triad of symptoms, namely, thrombocytopenia, eczema and immunodeficiency [28,29,30]; furthermore, they disrupt the activity of important functional domains, leading to more severe phenotypes [31,32]. nWASP was the second protein to be classified as a WASP family member due to the detection of several functional motifs shared with WASP. This 65 kDa protein was named neural WASP due to its shared homology with WASP and abundance in the brain, although it is widely expressed in different tissues throughout the body [33]. Under resting conditions, nWASP exists in an inactive, auto-inhibited confirmation whereby the main catalytic domain is shielded by the N-terminus regions. The WASP/WAVE family mediates the signals between the Rho GTPase family members, such as Rho, Rac and Cdc42, and the factors that modulate the actin cytoskeleton, in particular the actin-related protein 2/3 (Arp2/3) complex. Cdc42 can bind to the GBD domain, destabilising the folded confirmation of nWASP and exposing its catalytic domain [34,35]. Arp2/3 becomes activated when bound to the C-terminus CA region on WASP family proteins, allowing actin polymerisation to be initiated if an actin monomer is bound in conjunction with the V region [36,37,38,39].

Through its role as an organiser of the actin cytoskeleton, nWASP was also shown to be involved in the formation of membrane protrusions that are important for cell movement and its expression was shown to correlate with certain cancer phenotypes [34,40,41,42,43]. Consequently, nWASP has been recognised as a potential therapeutic target in a range of contexts, including wound healing.

The aims of this study were to validate nWASP as a novel therapeutic target for the treatment of chronic wounds by examining expression levels in human wound tissues. The therapeutic potential of targeting nWASP was then explored by examining the effect of inhibiting nWASP using the agents 187-1 and wiskostatin, which act to maintain nWASP in its inactive, auto-inhibited state (small-molecule inhibitors of nWASP, namely, wiskostatin and 187-1, bind to nWASP and allosterically block its activity by stabilising the closed, auto-inhibited conformation of nWASP. Both inhibitors act in the same way to stabilise the autoinhibited state of nWASP, blocking activation and hence actin polymerisation, but wiskostatin was used more extensively in research to examine the effects of nWASP activity. Wiskostatin is a cell-permeable N-alkylated carbazole that interacts with a cleft in the regulatory GBD of nWASP in the solution structure of the complex [44], as well as on human skin cell behaviour in vitro and in vivo on the closure of wounds in mouse models with impaired healing abilities. We also investigated the potential effects of these treatments on downstream signalling changes in skin cells in order to elucidate mechanistic effects.

## 2. Materials and Methods

### 2.1. Cell Lines and Culture Conditions

Human keratinocyte cells (HaCaT cells) from the German Cancer Institute/Cell Service, Germany, human vascular endothelial cells (HECV cells) from Interlab, Italy, and TE 354.T cells (LCG Standards, Bury, UK) were cultured in Dulbecco’s Modified Eagle Medium (DMEM) (Sigma-Aldrich, Dorset, UK) supplemented with 10% foetal bovine serum (FBS; Sigma-Aldrich, Dorset, UK), 100 units/mL penicillin and 100 µg/mL streptomycin (Sigma-Aldrich, Dorset, UK). The cells were incubated at 37 °C, 5% CO_2_ and 95% humidity.

### 2.2. Tissue Collection

Fresh tissues from chronic wounds (*n* = 14), acute wounds (*n* = 10) and normal skin from healthy volunteers (*n* = 10) were collected under the approval of the local ethical committee (ethical approval ID: 04/WSE03/92). Chronic wound tissues were obtained from patients with chronic leg ulcers that had been present for a minimum of 6 months prior to their biopsy with no evidence of healing 6 weeks before the biopsy. Acute wound tissues were obtained from patients with acute surgical wounds following the excision of pilonidal disease and normal tissues were from healthy, unwounded skin. A second cohort of chronic wound tissues was collected from chronic venous leg ulcer patients following ethical approval from a local committee (South East Wales Ethics Committee reference number 09/WSE02/51). Wound size was recorded at the time of the initial biopsy and again after 3 months, during which wounds were treated as per best medical practise (*n*-77). Wounds that reduced in size over this period were described as ‘healing/healed’ and those that grew or did not reduce in size were described as ‘non-healing’. All tissues were snap-frozen following collection and stored at −80 °C until processing. Written informed consent was obtained from each patient who agreed for a biopsy to be taken. Wound cohorts were previously described [45,46,47].

### 2.3. Tissue Processing for the RNA Extraction and Reverse Transcription

Tissues were sectioned on a cryostat (Leica, Microsystems Ltd., Milton Keynes, UK) with either a 7 µm thickness for immune-histochemical analysis or at 20 µm thickness for the extraction of RNA. Approximately 20 sections from the same patient sample biopsy were pooled and homogenised in an ice-cold TRI reagent (Sigma-Aldrich, Poole, UK) using a hand-held homogeniser (Cole Palmer, London, UK). RNA was extracted from the tissues following the manufacturer’s instructions, and the same protocol was also used to extract RNA from cultured cell lines. Samples were standardised and cDNA was subsequently generated (BioRad, Hemel Hempstead, UK).

### 2.4. Tissue Processing for the Ex Vivo Model

The *ex vivo* effects of the nWASP inhibitor 187-1 in chronic human wound tissues were examined using a method previously established [48]. Briefly, fresh biopsies from human chronic wounds were immediately placed in a buffer that mimics physiological fluid and contains a mixture of antibiotics. The tissues were finely minced using a sterile scalpel to sizes less than 1 mm in diameter. After extensive washing in the buffer, the living tissues were immediately embedded in an extracellular matrix gel. The gels and the topping solution included the treatments (nWASP inhibitor). The tissues were photographed daily. The degree of expansion from the implanted tissues was calculated using the imager, as previously reported [48].

### 2.5. Quantitative, Real-Time Polymerase Chain Reaction (qPCR) and Conventional PCR

Analysis of gene transcript expression was carried out using qPCR with cDNA produced from a human wound and skin tissues and conventional PCR using cDNA produced from RNA extracted from cell lines. This study adopted Ampliflor quantitation technology in which one set of gene-specific primers (designed using Beacon Design software, PREMIER Biosoft, Palo Alto, CA, USA) and Uniprimer probes (Intergen Inc., New York, NY, USA) were used. The reaction was carried out using the ICycleIQ (BioRad, UK). The real-time qPCR conditions were 95 °C for 15 min, followed by 60 cycles of 95 °C for 20 s, 55 °C for 30 s and 72 °C for 20 s. In all the assays, GAPDH and actin were amplified and used as housekeeping controls, and an internal standard was also employed for quantitation purposes. The nWASP primers were as follows: BNDF (F8: TTCATACTTTGGTTGCATGA, R8: TTCAGTTGGCCTTTTGATAC), GAPDH (F8: GGCTGCTTTTAACTCTGGTA, R8: GACTGTGGTCATGAGTCCTT), nWASP (F8: AGTCCCTCTTCACTTTCCTC, R8: GCTTTTCCCTTCTTCTTTTC), TrkB (F2a: CCCACTCACATGAACAATGG, R2a: TCAGTGACGTCTGTGGAAGG), Actin (F8: GGACCTGACTGACTACCTCA, zR8: ACTGAACCTGACCGTACAAGCTTCTCCTTAATGTCACG), BDNF (F8: TTCATACTTTGGTTGCATGA, Rz8: ACTGAACCTGACCGTACACTCTTGAACCTGCCTTGG), GAPDH (F: CTAGTACGTCGTGGAGTC, zR: ACTGAACCTGACCGTACACAGAGATGATGATGACCCTTTTG), PDPL (F: GAATCATCGTTGTGGTTATG, zR: ACTGAACCTGACCGTACACTTTCATTTGCCTATCACAT) and nWASPKD (F8: AGTCCCTCTTCACTTTCCTC, zR8a: ACTGAACCTGACCGTACAAGATCTCTGTGGATTGTCCT).

### 2.6. Reagents and Treatments

nWASP inhibitors, wiskostatin (Merck Pharmaceuticals, Watford, UK) and 187-1 (TOCRIS, Bristol, UK) were used. For the in vitro studies, wiskostatin was dissolved in 30% dimethyl sulfoxide (DMSO, Sigma-Aldrich, Dorset, UK) in normal cell culture medium to a stock concentration of 300 µM, whereas 187-1 was diluted in BSS. For the in vivo studies, nWASP inhibitor compounds, 187-1 and wiskostatin were formulated for systemic and topical application. For systemic application, 187-1 was dissolved and diluted in BSS to the required concentration. Wiskostatin was first dissolved in DMSO (Sigma-Aldrich, Gillingham, UK) at a concentration of 5 mg/mL. The DMSO solution was then gradually diluted in BSS in order to avoid precipitation. The solutions were prepared so that each 100 µL contained the correct amount of compounds and was stored at −20 °C until used. The primary antibodies used were as follows: actin (sc-16515, Insight Biotechnology, Middlesex, UK), Erk1/2 (v114A, Promega, Southampton, UK), GAPDH (sc32233, Insight Biotechnology, Middlesex, UK), PLCγ1 (sc81, Insight Biotechnology, Middlesex, UK), p-PLCγ1 (sc22141, Insight Biotechnology, Middlesex, UK), nWASP (NBP1-82512, Novus Biologicals, Abingdon, UK), TrkB Y816 (ABN1381, EMD-Millipore, Watford, UK), TrkB pan (07-225, EMD-Millipore, Watford, UK) and TrkB pan (sc-136990, Insight Biotechnology, Middlesex, UK). The secondary antibodies were as follows: goat anti-mouse IgG (A4416, Sigma-Aldrich, Dorset, UK), goat anti-rabbit IgG (A6154, Sigma-Aldrich, Dorset, UK), rabbit anti-goat IgG (A5420, Sigma-Aldrich, Dorset, UK), DAPI (D1306, Life Technologies, Warrington, UK), anti-rabbit AlexaFluor 488 (A21206, Life Technologies, Warrington, UK), anti-mouse AlexaFluor 488 (A21202, Life Technologies, Warrington, UK), anti-rabbit AlexaFluor 594 (A21207, Life Technologies, Warrington, UK), anti-mouse AlexaFluor 594 (A21203, Life Technologies, Oxford, UK) and anti-goat AlexaFluor 594 (A11058, Life Technologies, Warrington, UK).

### 2.7. Functional Assays

#### 2.7.1. Electric Cell–Substrate Impedance Sensing (ECIS)

ECIS instruments (Applied Biophysics Inc., New York, NJ, USA) were used to electrically monitor the coverage of gold electrodes by cells by measuring the resistance and impedance at a frequency of 4 kHz unless otherwise specified. Cells were seeded in a culture medium containing treatments. Cells were then constantly monitored following seeding and electrical wounds were applied where described. Wound settings were a time of 30 s, current of 2400 μA and frequency of 60,000 Hz. Increased resistance and impedance illustrate a greater number of cells present on the electrode. This can therefore be used to assess attachment and motility.

#### 2.7.2. In Vitro Cell Viability Test

Briefly, cells were seeded in a 96-well plate in varying treatment concentrations/controls and incubated for 92 h. After a further 4 h incubation in 1:10 MTT (Thiazolyl Blue Tetrazolium Bromide, Sigma-Aldrich, Gillingham, UK) solution (5 mg/mL in phosphate buffered solution (PBS)), acidified isopropanol (Sigma-Aldrich, Dorset, UK) was applied and the optical density (OD) was determined using an absorbance reader (Biotek ELx800, Swindon, UK) at 540 nm and used to calculate relative cell viability at each treatment concentration [49].

#### 2.7.3. In Vitro Growth Assay

Cells were seeded into a 96-well plate with appropriate treatments and fixed after 1/2/4 day incubation periods using 4% formalin (Sigma-Aldrich, Dorset, UK). Plates were stained with 1% crystal violet (Sigma-Aldrich, Gillingham, UK), which was then extracted from cells using 10% acetic acid (Sigma-Aldrich, Dorset, UK) in distilled water (*v*/*v*). Absorbance was determined at a 540 nm wavelength on an absorbance plate reader (Biotek ELx800, Swindon, UK).

#### 2.7.4. In Vitro Carrier Bead Assay

A total of 100 μL of Cytodex-2 carrier beads (Sigma-Aldrich Ltd., Gillingham, UK) were added to 10 mL of 1 × 10^4^ cells/mL and gently mixed. The cells were then left at 37 °C for 2 h. Following a wash with medium, cells were allowed to settle until aliquoted into a 96-well plate and treated with inhibitors. After incubation overnight, the beads were washed off in PBS and the cells that had migrated onto the culture vessel floor were counted after visualisation with crystal violet.

#### 2.7.5. In Vitro Scratch Assay

Cells were seeded in appropriate treatments into each well on a 24-well plate. Upon reaching confluence, the monolayer was scratched to create a linear wound. The plate was placed in an EVOS^®^ FL Auto Imaging System (Life Technologies, Oxford, UK), which maintained the plate in normal culture conditions throughout the experiment. Images were captured of the wound every 30 min for up to 24 h.

#### 2.7.6. In Vitro Adhesion Assay

Wells were pre-coated with Matrigel basement membrane matrix (BD Biosciences, Oxford, UK) at 50 μg/mL in a normal culture medium. Cells were then seeded into each well onto the Matrigel membrane in treatments and incubated for 25 min. Adherent cells were fixed, stained and quantified as above.

#### 2.7.7. Tubule Formation Assay

A Matrigel matrix (10 mg/mL) was gently pipetted into the bottom of each well of a 96-well plate. The plate was placed at 37 °C for 40 min whilst the Matrigel polymerised. Then, 4 × 10^4^ cells (and treatments/control reagents where appropriate) were plated on top of the Matrigel in each well in triplicate and incubated at 37 °C, 5% CO_2_ and 95% humidity. Images of each well were captured at X5 magnification and intervals ranging from 30 to 1170 min using a Leica DMi1 microscope equipped with an MC120 HD camera and Leica Application Suite version 3.0.0 software (Leica Microsystems, Milton Keynes, UK). Analysis of the images was carried out using ImageJ, where the lengths of cell structures that form part of a tubule structure were measured to track the progress of tubule formation.

### 2.8. In Vivo Tolerance Test

The main tolerance tests were conducted using CD-1 athymic mice (Charles River Laboratories, Oxford, UK), owing to their slow and steady rate of growth and the ease of observing changes in the skin (hairless) and other possible side effects. Briefly, CD-1 mice that were 4–6 weeks old and 20 g in weight were housed in filter-topped cages. 187-1 (MW 1784, dissolved in BSS buffer) and wiskostatin (MW 426, dissolved in DMSO and diluted in BSS) were injected via the intraperitoneal route on a daily basis. Dosages administered were 1 and 10 µM in 100 µL volumes for each compound, equivalent to 1.8 g/kg/day and 17.8 g/kg/day for 187-1 and 0.43 g/kg/day and 4.3 g/kg/day for wiskostatin. CD-1 mice were observed daily and weighed twice weekly based on the previous in vitro experiments. An additional tolerance and efficacy test was carried out using the diabetic db/db strain obtained from Harlan Laboratories (Cambridge, UK), which exhibited impaired wound healing abilities. These mice were 4–6 weeks old and when their body weight reached 20 g they were used for tolerance and efficacy tests.

### 2.9. In Vivo Efficacy Test and Wound Healing

An ear punch method previously described [50] was used to create wounds in the mice that were 1 mm in diameter. Treatment was given systemically or topically. For topical application, two carrier gels that are currently used in wound care were used. The inhibitors were diluted from the master stock in the gel at a concentration of 1 mg/g followed by low-speed homogenisation using a hand-held homogeniser. For use, small amounts (150 µL) of the gel were rubbed into the wound area. Both treatments were given every other day. For systemic application, 187-1 was applied at 0.5 and 5 µM (equivalent to 0.89 g/kg/day and 8.9 g/kg/day) and wiskostatin was applied at 1 and 10 µM (equivalent to 0.43 g/kg/day and 4.3 g/kg/day). Images were obtained weekly. The sizes of the wounds were determined using image analysis software. Data are presented in two ways: as the area of the wounds in pixels, where two sample Student t-tests were used for statistical analysis, or as the change in the size of the wound from the starting point calculated using the following formula: (area at a given point—area at the starting point)/(area of the starting point) × 100, in which case, the Bonferroni model was used for data analysis.

### 2.10. Microarray Analysis

This study used the Kinexus^TM^ KAM880 protein array service provided by Kinexus Bioinformatics Ltd. (Vancouver, BC, Canada). Signal quantification was performed with ImaGene 8.0 by Kinexus Bioinformatics Ltd., which has predetermined settings for spot segmentation and background correction. The background-corrected raw intensity data were then globally normalised by summing the intensities of all the net signal median values for a sample to obtain the globally normalised signal intensities for each protein. The percentage change of the treated samples from the control was calculated based on the globally normalised intensity for each protein using the following calculation: %CFC = (globally normalised treated − globally normalised control)/globally normalised control) × 100. The percentage error range was also calculated to examine how tightly the globally normalised net signal intensity varied for duplicate spots of the same protein in the sample. The z-scores were also calculated by subtracting the overall average intensity of all replicate spots from the raw intensity for each spot and then dividing it by the standard deviation of all the measured intensities within each sample. The z-ratio was calculated by dividing the differences between the observed z-scores by the standard deviation of all the differences for that comparison. Several factors were used to determine the most important changes in protein expression and phosphorylation including %CFC, error ranges, value for the globally normalised intensity of one of the samples of >1500 and significance based on z-ratios of <−1.64 or >1.64.

### 2.11. Immunofluorescence

Cells were cultured in Millicell EZ 8-well chamber slides (Merck Millipore, Watford, UK). To fix the cells, the culture medium was removed and the cells were washed with PBS and then fixed in 100% ice-cold ethanol. To proceed with immunofluorescence staining, the cells were washed 3 times in PBS and then permeabilised using 0.1% Triton X-100 (Sigma-Aldrich, Dorset, UK). Blocking buffer (5–10% donkey serum (D9663, Sigma-Aldrich, Dorset, UK)) in PBS was added to each well. The slide was left for 3 h at room temperature in a blocking buffer. The chamber slide was then incubated with primary antibodies for an hour on the bench or at 4 °C overnight. Secondary antibodies were (1:500) with the addition of DAPI (1:100), and each primary antibody was incubated with the corresponding secondary for a further hour. Following three washes in PBS, the slides were mounted in FluorSave™ (Calbiochem, Nottingham, UK) and allowed to dry before being visualised using an Olympus BX51 microscope with a Hamamatsu Orca ER digital camera at ×40. Images were analysed using ImageJ.

### 2.12. Immunohistochemistry (IHC)

Immunohistological analysis using an avidin–biotin peroxidase technique was performed on human tissue samples collected from cohort 2. The frozen sections were fixed in dried acetone (10162180, Fisher Scientific, Loughborough, UK), air-dried and washed in PBS. Sections were then incubated in 0.1% BSA/10% horse serum in PBS (referred to as blocking solution) in a humidified box at room temperature, followed by primary antibody solutions (diluted in blocking buffer to a final concentration of 2 µg/mL). Following washing with PBS, biotinylated horse anti-mouse/rabbit IgG secondary antibody (Vector Laboratories, Oxford, UK) was applied for 30 min, followed by ABC reagent for 30 min, both of which were provided in the VECTASTAIN^®^ Elite ABC Kit (Vector Laboratories, Peterborough, UK). 3,3′-Diaminobenzidine (DAB)(Sigma-Aldrich, Gillingham, UK) substrate (5 mg/mL) was used to develop the final reaction and the sections were counterstained with Gill’s haematoxylin (Vector Laboratories, Oxford, UK). Following dehydration and clearing in xylene, sections were mounted in Distyrene Plasticizer Xylene (DPX, Merck Pharmaceuticals, Gillingham, UK). Staining was visualised using a Leica DM1000LED microscope with an MC120 HD camera and Leica Application Suite (version 3.0.0) software (Leica Microsystems, Milton Keynes, UK). The localisation and intensity of staining were judged blindly by two people independently. Positive staining was seen as a brown/black deposit, whilst negatively stained cells could be clearly distinguished using a blue nucleated stain.

### 2.13. Protein Extraction, SDS-PAGE and Western Blotting

A lysis buffer was used to extract protein from cells, which was then used for SDS-PAGE. Proteins were transferred onto Immobilon^®^ PVDF membranes (Merck Millipore, Watford, UK), which were blocked and probed with primary antibodies and then incubated with the corresponding peroxidase-conjugated secondary antibodies. Proteins were visualised using an EZ-ECL Kit (Biological Industries, Beit Haemk, Israel).

### 2.14. siRNA Silencing of nWASP

nWASP siRNA (sc36006) was obtained from Insight Biotechnology (UK) and non-targeting siRNA (NT) was obtained from Dharmacon (D001810; Layfayette, CO, USA). Cells were transfected with nWASP siRNA (sc360006; Insight Biotechnology, UK) at varying concentrations or NT control siRNA at the same concentration, as described. siRNA, which was diluted according to the desired end concentration in SFM, was combined with equal volumes of Lipofectamine 3000 reagent (ThermoFisher Scientific, Boston, MA, USA). This siRNA/Lipofectamine 3000 mix in SFM (with no antibiotics) was allowed to stand at room temperature for 30–40 min. Antibiotic-free DMEM supplemented with 5% FBS was then added to the siRNA/Lipofectamine 3000 solution to achieve the desired end concentration of reagents and make up the required volume for application to cells; then, the final solution was gently applied to each well. Cells were incubated under normal culture conditions and a normal culture medium was used 24 h after transfection for any further culturing or assays unless stated otherwise.

### 2.15. Statistical Analysis

Statistical analysis was conducted using Minitab, SPSS, GraphPad 6.0 Prism (PRISM, Boston, MA, USA) and an online chi-square service tool. Transcript levels from qPCR experiments are reported as median ± SEM and the Mann–Whitney test was used to analyse qPCR data. Representative data are presented. A *p*-value < 0.05 was considered statistically significant.

## 3. Results

### 3.1. nWASP Expression in the Human Chronic Wound Tissues and Cell Lines

**Figure 1:**
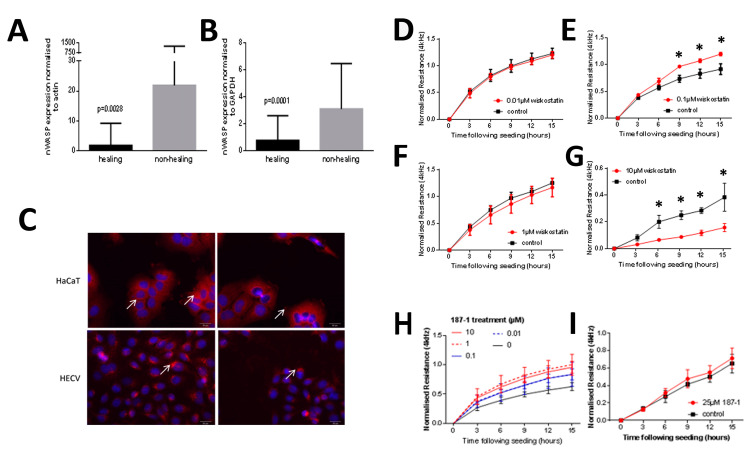
**Analysis of nWASP in tissues and cells with the effect of the inhibitor treatment on the cell attachment.** qPCR analysis of wound tissues from cohort 1 (**A**) and cohort 2 (**B**). Immunofluorescence staining for nWASP in keratinocyte and endothelial cell lines; the scale bar is 100 µm (**C**). The effect of wiskostatin on HaCaT attachment and spreading, as measured using ECIS (**D**–**G**). The effect of 187-1 treatment on HaCaT attachment and spreading, as measured using ECIS (**H**,**I**). * indicates statistical difference (*p* < 0.05).

qPCR analysis of human chronic wound tissues demonstrated that nWASP transcript expression was significantly increased in the non-healing chronic wounds compared with those defined as healing in the 12 weeks following biopsy collection. In cohort 1, nWASP expression normalised with actin in healing/healed wounds (*n* = 20) was significantly lower than in non-healing wounds (*n* = 49) (*p* = 0.0028) (Figure 1A). Likewise, cohort 2 showed a similar trend where the nWASP transcript levels were significantly lower (*p* = 0.0001) in the healing chronic wounds (*n* = 77) than in the non-healing chronic wounds (*n* = 32) (Figure 1B). nWASP protein expression was examined in two human cell lines, representative of typical skin cells, HaCaT keratinocyte cells and HECV vascular endothelial cells (Figure 1C).

### 3.2. The Effect of nWASP Inhibition on the HaCaT Cell Attachment and Spreading

Initial ECIS experiments were carried out over a range of inhibitor concentrations. At 0.01 µM, no change was observed (Figure 1D). However, at 0.1 µM levels, significantly higher resistances were detected in the wiskostatin-treated cells compared with the control (Figure 1E). This increase in resistance did not continue with increased inhibitor treatments (Figure 1F,G). At high levels of wiskostatin, the opposite effect was found (Figure 1G). The ECIS analysis of HaCaT cells treated with a range of concentrations of 187-1 demonstrated that the resistance at 4 kHz was significantly increased with the inhibitor between concentrations of 0.01–10 µM 187-1 (Figure 1H). The increase in resistance compared with the control in the 15 h following seeding was not found with a higher level of 187-1 at 25 µM (Figure 1I).

### 3.3. ECIS Analysis of nWASP Knockdown in HaCaT Cells

**Figure 2:**
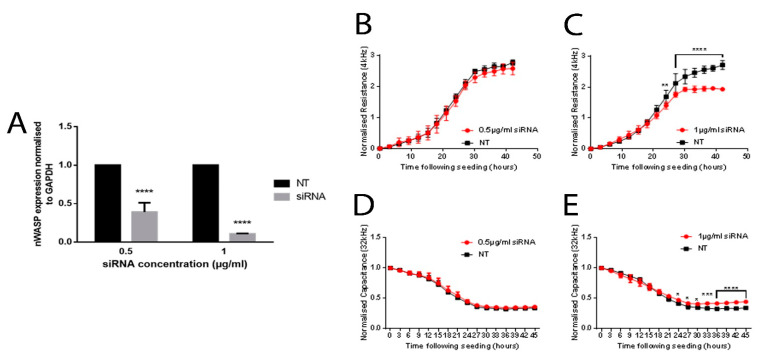
**nWASP knockdown in the HaCaT cells.** qPCR and PCR analysis of nWASP transcript expression (**A**). ECIS analysis of nWASP knockdown HaCaT cells. HaCaT cells treated with nWASP siRNA (**B**–**E**). * indicates statistical significance (* *p* < 0.05, ** *p* < 0.01, *** *p* < 0.001 **** *p* < 0.0001).

nWASP was knocked down using siRNA, with a successful knockdown shown in Figure 2A. There was no difference in the resistance or capacitance in cells treated with the lower concentration of siRNA (0.5 µg/mL) (Figure 2B,D). A significant difference in the resistance and capacitance was found between the cells treated with 1.0 µg/mL siRNA and the NT cells from the point where the measurements reached a plateau, at approximately 30 h from seeding, and the cells were assumed to have reached confluency (Figure 2C,E).

### 3.4. Inhibition of Growth following the nWASP Inhibitor Treatment

**Figure 3:**
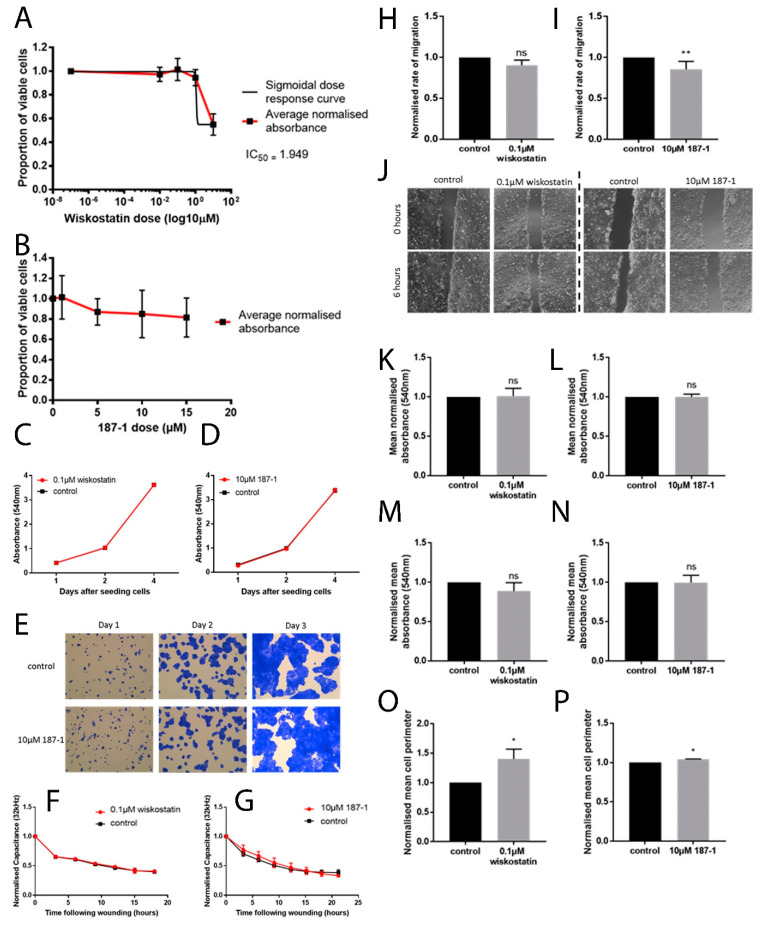
**The effect of nWASP inhibition on the HaCaT cell behaviour.** Dose–response curves (**A**,**B**). HaCaT cells treated with 0.1 µM wiskostatin (**C**) or 10 µM 187-1 (**D**). Average absorbance ± SD is presented, representative data are shown and *n* = 5 replicates were in each independent repeat. Representative images of crystal-violet-stained cells (**E**). HaCaT cells following an electrical wounding treated with 0.1 µM wiskostatin (**F**) or 10 µM 187-1 (**G**). The effect of wiskostatin and 187-1 on the rate of migration of HaCaT cells: 0.1 µM wiskostatin (**H**) and 10 µM 187-1 (**I**). Normalised rate of migration + SD is presented. Representative images of the scratch wound at 0 and 6 h are also shown (**J**). The 0.1 µM wiskostatin (**K**) and 10 µM 187-1 (**L**) treatment results are shown. The change from the control is presented with the mean + SD shown. The effects of 0.1 µM wiskostatin (**M**) and 10 µM 187-1 (**N**) on the normalised absorbance, and the effects of 0.1 µM wiskostatin (**O**) and 10 µM 187-1 (**P**) on the spread of HaCaT cells. * indicates statistical significance from 0 h to each subsequent time point (* *p* < 0.05, ** *p* < 0.01, ns: not significant).

No significant inhibition in growth was found following treatment with the full range of 187-1 tested and hence no IC_50_ value was calculated (Figure 3A,B). There was also little difference in the control and inhibitor-treated cells treated with 0.1 µM wiskostatin and 10 µM 187-1 (Figure 3C–E, respectively).

### 3.5. The Effect of nWASP Inhibition on the Keratinocyte Cell Migration and Adhesion

Several methods were utilised to examine the effect of nWASP inhibitors on cell motility/migration. Where HaCaT cells were treated with 0.1 µM wiskostatin, the rate of migration assessed using ECIS was found to be, on average, 90% of the control when treated with wiskostatin (*p* = 0.0572; Figure 3F–H). In 187-1 treated cells, the rate of migration was found to be significantly decreased (*p* = 0.0234) at 85% of the control migration rate (Figure 3I,J). When using a micro-carrier bead assay, there were no observable differences after the treatment (Figure 3K,L). The effect on the ability of HaCaT cells to initially adhere to a Matrigel matrix in response to the nWASP inhibitor treatment showed no significant difference in the number of cells adhered to the matrix following either the 0.1 µM wiskostatin treatment or the 10 µM 187-1 treatment (Figure 3M,N). An alternative measure of cell adhesion through measuring the spread of HaCaT cells several hours after seeding in nWASP inhibitor treatments was used. The spread according to the perimeter of cells 6 h after seeding was found to be significantly increased in the cells treated with 0.1 µM wiskostatin compared with the controls (*p* = 0.0137, Figure 3O). The cell perimeter was also found to be increased in cells treated with 10 µM 187-1 compared with the controls, but this increase was very small (*p* = 0.01, Figure 3P).

### 3.6. The Effect of nWASP Inhibition on the HECV Cell Behaviour

**Figure 4:**
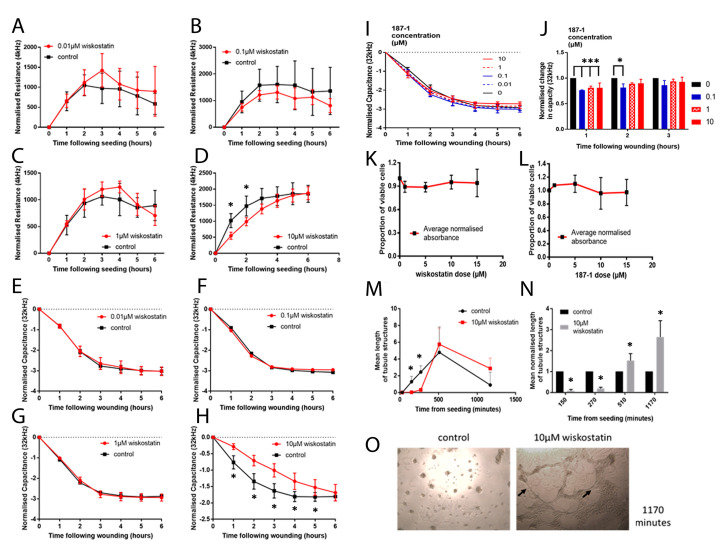
**The effect of wiskostatin on the HECV cell behaviour.** HECV cells were treated with wiskostatin at 0.01, 0.1, 1 and 10 µM concentrations (**A**–**D**). HECV cells were treated with wiskostatin at 0.01, 0.1, 1 and 10 µM concentrations (**E**–**H**). HECV cells were treated with 187-1 at 0, 0.1, 1 and 10 µM concentrations and monitored for 6 h following an electrical wounding event (**I**). The mean changes in capacity in the 187-1-treated wells are also shown (**J**). Following 48 h with wiskostatin and 187-1 (**K**,**L**). Mean lengths of the tubule structures of 10 µM wiskostatin-treated and control HECV cells (**M**). Tubule structure lengths at time points of 150–1170 min (**N**,**O**) in terms of the number of pixels. * indicates statistical significance (* *p* < 0.05, *** *p* < 0.001).

The effect of the wiskostatin treatment was initially examined using ECIS (Figure 4A–C). The 10 µM wiskostatin treatments significantly delayed the formation of a HECV monolayer, as shown by the decrease in the resistance in the 10 µM wiskostatin treated cells (Figure 4D). Wiskostatin caused a significant reduction in the resistance of the HECV cells compared with the control during the initial attachment and spreading phase following seeding as the resistance increased prior to reaching a plateau. This significant reduction in the resistance was also found in the HECV cells treated with higher levels of the inhibitor (1 and 10 µM). Low levels of the inhibitor did not have a significant effect on the electric wounding (Figure 4E–G). However, at 10 µM wiskostatin, the capacitance was consistently significantly increased, suggesting a delay in the healing of the electrically wounded inhibitor-treated cells (Figure 4H). The 187-1 treatment at 0.1, 1 and 10 µM concentrations caused the capacitance at 32 kHz to consistently decrease (Figure 4I,J). The inhibitors did not appear to affect the growth of the HECV cells, as no change in the growth of more than 12% was observed (Figure 4K,L). The lengths of the cell structures that formed part of microtubules in the tubule formation assay were measured following seeding and the mean total length for each image was calculated (Figure 4M). The wiskostatin treatment significantly inhibited the formation of tubule structures immediately following seeding but did not prevent the formation of tubule structures, which typically developed after about 8 h. Following the maturity of these tubule formations, the HECV cells treated with the control medium began to cluster and tubule formations were lost. However, in the wiskostatin-treated cells, although the tubules became thicker and some clustering was also evident, tubule formation was still evident after 20 h (Figure 4N). There was a significant decrease in the tubule structures in the wiskostatin-treated cells following seeding with a subsequent significant increase in tubule structures after 8 h, where the tubule structures were maintained in wiskostatin treatment and lost in the control cells as the HECV cells formed clusters (Figure 4O).

### 3.7. The 187-1 and Wiskostatin In Vivo Effects

**Figure 5:**
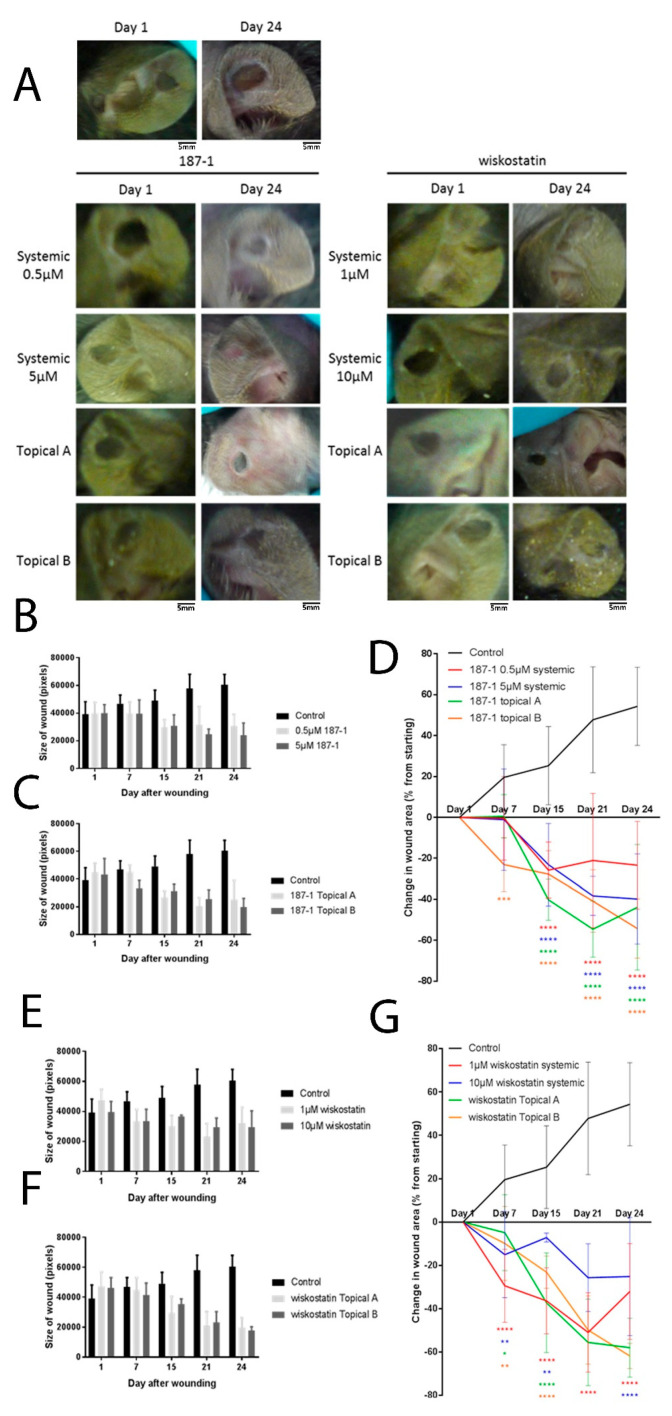
**nWASP inhibition and the change in healing rates in a murine mouse model.** The effect on the size of the hole punch wound in pixels (mean +/− SD, *n* = 6) following the systemic (**A**) and topical (**B**) application of 187-1. Percentage changes in wound area from the first measurement after wounding (day 1) (**C**). The effect of the administration of wiskostatin is shown in (**D**–**G**). * indicates statistical significance (* *p* < 0.05, ** *p* < 0.01, *** *p* < 0.001 **** *p* < 0.0001).

The 187-1 and wiskostatin were delivered systemically on a daily basis as part of a tolerance assay for two weeks in athymic CD-1 and db/db mouse strains. Two concentrations, namely, 0.5 and 5 µM of 187-1, were given systemically (interperitoneal injections daily in a volume of 0.5 mL; mice ears were photographed twice weekly and weighed to assess health, together with general health and appearance). Following this, 5 mm hole punches were made in each ear and treated twice weekly, either with a topical injection (ears massaged twice weekly or interperitoneal injections as per tolerance testing). Healing of the wounds was significantly increased after the systemic and topical administration of 187-1 (Figure 5A,B). After 24 days, the wounds treated with 0.5 and 5 µM 187-1 were significantly smaller than the control group (*p* < 0.0001; Figure 5C). The topical application of 187-1 in both carrier gels A and B also showed a significant effect after 24 days (*p* < 0.0001). The 187-1 in topical carrier gel B appeared to encourage wound healing at a faster rate compared with the other treatments, as the wound size was significantly smaller than the control group after only 7 days, whereas the other treatments saw a significant difference after 15 days. Two concentrations of wiskostatin (1 and 10 µM) were also given systemically. Healing of the wounds was increased following both methods of administration of wiskostatin after 24 days (Figure 5D,E). After 24 days, the wounds treated with 1 and 10 µM wiskostatin were significantly smaller than the control group (*p* < 0.0001; Figure 5F). The topical application of wiskostatin in both carrier gels A and B also showed a significant effect after 24 days (*p* < 0.0001). The wiskostatin treatment appeared to encourage healing at a faster rate than the 187-1, as a significant reduction in the wound size compared with the control wounds was observed in all the treatment methods after only 7 days from wounding.

### 3.8. Protein Signalling Changed following the Wiskostatin Treatment

**Figure 6:**
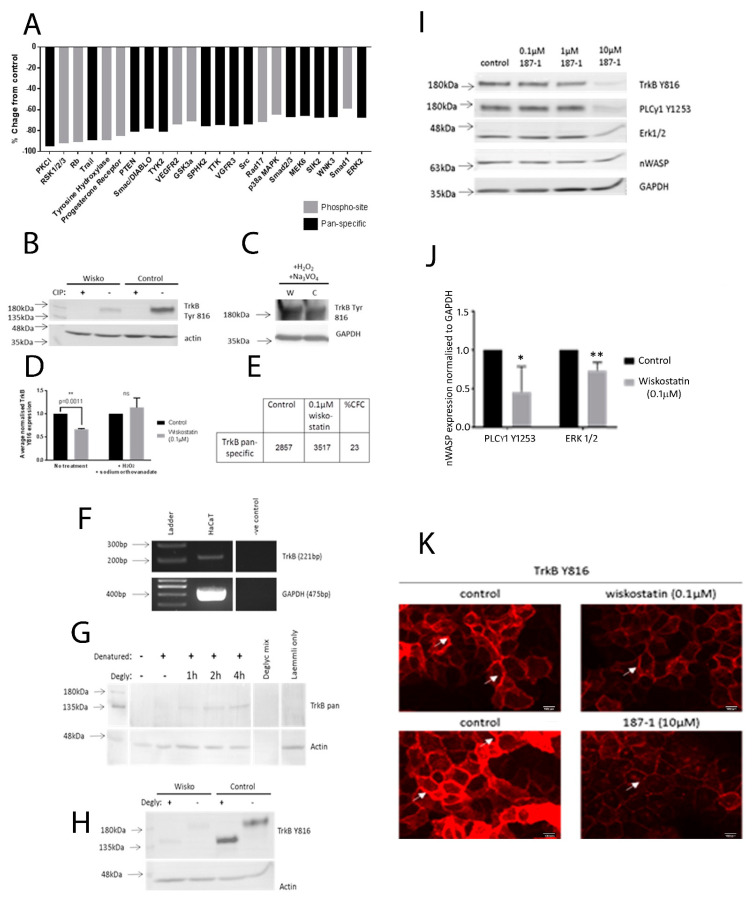
**Protein signalling changes in response to the wiskostatin treatment.** (**A**) Changes in the total protein expression or phosphorylation sites in response to the 0.1 µM wiskostatin treatment for 1 h according to the %CFC. Protein samples that were treated with CIP at 0.6 units/µg for 1 h (**B**). Positive controls (**C**). Integrated densities as a change from the control (**D**). Globally normalised expression of total TrkB protein and %CFC according to protein array analysis (**E**). TrkB transcript expression in the HaCaT cell line (**F**). Protein extracts from HaCaT cells were treated with denaturation and deglycosylation reagents (**G**). Samples from cells treated with 0.1 µM wiskostatin/control were denatured and then deglycosylated to examine the TrkB Y816 activity (**H**). Western blot analysis of TrkB Y816, PLCγ1 Y1253 and Erk1/2 of HACAT cells treated with 0.1 µM wiskostatin or 10 µM 187-1 (**I**,**J**). Immunofluorescence analysis of TrkB Y816 expression in HaCaT cells serum starved for 4 h and treated with nWASP inhibitors; scale bar indicates 50 µm (**K**). * indicates statistical significance (* *p* < 0.05, ** *p* < 0.01,.ns: not significant).

A protein array analysis was carried out to examine the protein signalling changes that took place in HaCaT cells in response to the nWASP inhibitor treatment. The most significantly decreased protein signals based on the z-ratio are presented in Figure 6A. A full description of the changes in protein expression or phosphorylation of the most significantly increased or decreased signals (i.e., −1.64 < z-ratio < 1.64) can be found in Table 1. Amongst the most significantly increased or decreased proteins, several membrane-bound proteins and receptors were identified and are highlighted in grey. Through further experiments to examine the protein signalling changes following the 0.1 µM wiskostatin treatment, TrkB signalling was found to be significantly affected by inhibitor treatment, as described in the following sections.

Matched Western blot analysis using protein extracts from HaCaT cells highlighted that TrkB phosphorylation at tyrosine 816 (Y816) was significantly downregulated in the wiskostatin-treated samples (*p* = 0.0011, *n* = 3; Figure 6B). This signal was lost in the samples treated with CIP, a reagent which removes phosphorylation from protein samples, whereas no effect on total protein expression was seen in the actin-positive control bands. This demonstrated that this signal, which was detected using the ABN1381 TrkB Y816 antibody, was a phosphorylation signal, as intended. Furthermore, no significant difference between the TrkB Y816 expression in samples from wiskostatin and control-treated cells that were exposed to hydrogen peroxide and sodium orthovanadate was detected (Figure 6C). This suggested that the differential expression of TrkB Y816 that was detected between wiskostatin and control-treated samples was due to a change in the phosphorylation of TrkB at tyrosine 816 rather than a change in total protein. The TrkB total protein was found to have a 23% CFC with a z-ratio of 0.27, which was not deemed significant (Figure 6E). The change in TrkB Y816 expression in the wiskostatin-treated cells from the controls shown in Figure 6D demonstrates that the tyrosine 816 phosphorylation of TrkB was affected by wiskostatin treatment.

### 3.9. TrkB Was Expressed in the HaCaT Cells and Was Glycosylated

In order to explain the larger-than-expected band observed in the Western blot results, experiments were carried out to validate whether TrkB was expressed in the HaCaT cell line and then to explore whether the TrkB protein may have existed in a glycosylated state. TrkB was found to be expressed in HaCaT cells at the transcript level, as shown in Figure 6F. Further experiments were carried out to determine why the observed band size for TrkB Y816 was at 180 kDa, higher than expected based on the literature, and why no corresponding band size could be seen using TrkB-pan antibodies. HaCaT protein samples were treated with deglycosylation reagents for 0–4 h and then Western blotting using TrkB-pan antibodies was carried out (Figure 6G). After 1 h of deglycosylation treatment, TrkB expression could be detected at 140 kDa using a TrkB-pan antibody, whereas no signal could be detected without deglycosylation treatment. This signal was not detectable using the usual, previously described protein extraction and denaturation techniques, which did not include a deglycosylation step. This experiment showed that TrkB was in fact expressed in HaCaT cells but was not previously detected using the ABN6180 TrkB antibody due to glycosylation, which may have blocked access of the antibody to the target site. Having shown that TrkB was present in HaCaT cells with a size of 140 kDa following deglycosylation, it was hypothesised that the TrkB Y816 signal should shift size from 180 kDa to 140 kDa after deglycosylation. Protein samples collected from HaCaT cells treated with 0.1 µM wiskostatin/vehicle control were denatured and treated with deglycosylation reagents for 1 h and then probed for TrkB-Y816 and actin as a positive control. Following the deglycosylation treatments, there was an obvious shift in the size of the protein detected using the TrkB-Y816 antibody from 180 kDa to 140 kDa (Figure 6H). No significant change in the level of phosphorylation of Y816 was observed following deglycosylation but the reduced phosphorylation at Y816 in the wiskostatin-treated cells compared with the control was still apparent, demonstrating that TrkB was glycosylated in the HaCaT cells’ larger protein band.

### 3.10. The TrkB Signalling Pathway Was Affected by Confluency, Serum Availability and nWASP Inhibitor Treatment in the HaCaT Cells

Having identified that wiskostatin treatment can alter the phosphorylation of TrkB at tyrosine 816 in HaCaT cells, an investigation into other protein signals that are related to the TrkB pathway was carried out. The effect of the additional nWASP inhibitor 187-1 over a range of concentrations (0, 0.1, 1 and 10 µM) on TrkB phosphorylation and signalling was also examined (Figure 6I). The effect on PLCγ1 phosphorylation at tyrosine 1253 and Erk1/2 following 1 h of 0.1 µM wiskostatin or a range of 187-1 treatments after 4 h serum starvation mirrored those observed at the TrkBY816 site in that the nWASP inhibitor treatment significantly decreased the expressions of TrkB Y816, PLCγ1 Y1253 and Erk1/2 (Figure 6H). This effect on TrkB Y816 signalling was only observed at the 10 µM 187-1 concentration. No effect on nWASP expression was found. PLCγ1 Y1253 and Erk1/2 expressions were significantly decreased in the wiskostatin-treated cells (Figure 6J). No significant difference in the PLCγ1 Y1253 expression between the wiskostatin and control cells was seen (Figure 6J). This suggested that the differential expression of PLCγ1 Y1253 that was detected between the wiskostatin-treated and control samples was due to a change in the phosphorylation status rather than a change in the total protein. Immunofluorescence studies confirmed the effect of nWASP inhibitors (Figure 6K).

### 3.11. BDNF Was Linked to TrkB Signalling and Functional Changes in the HaCaT Cells

**Figure 7:**
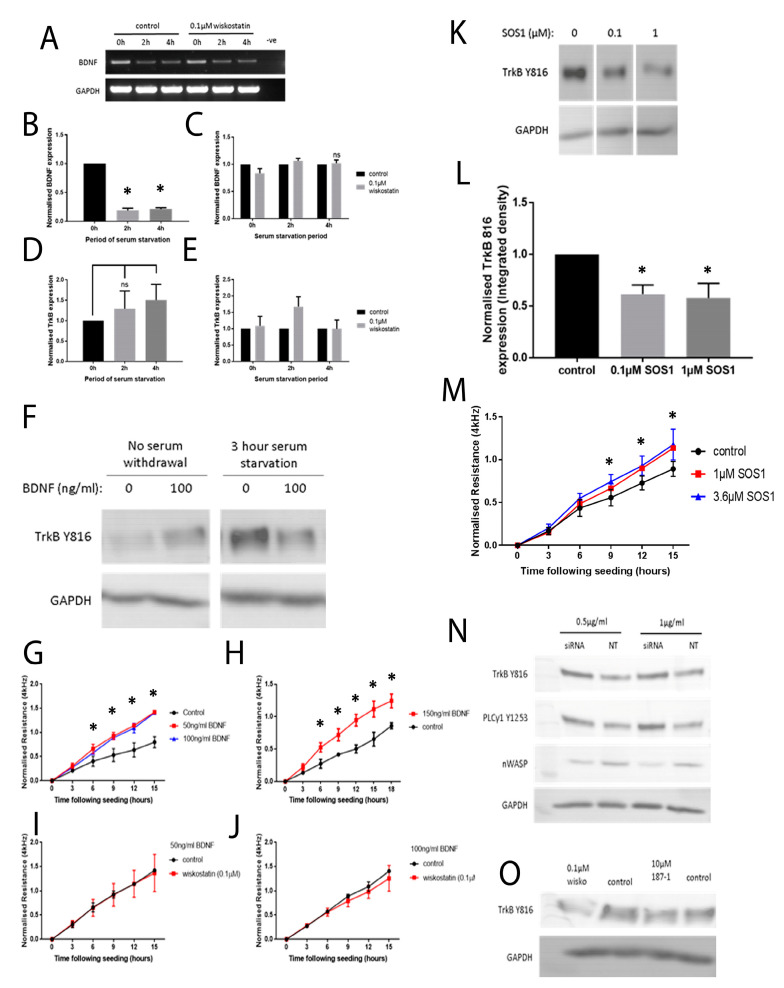
**BDNF and TrkB transcript expression in the HaCaT cells and the effects of the BDNF, SOS1 and knockdown models.** PCR/qPCR results for BDNF and TrkB expression in HaCaT cells treated with 0.1 µM wiskostatin (**A**,**B**); wiskostatin-treated samples at each serum starvation time point (**C**). qPCR analysis results of the TrkB transcript expression (**D**) and controls (**E**). Western blot analysis of TrkB Y816 (**F**). HaCaT cells were treated with rhBDNF at 50 and 100 ng/mL (**G**) and 150 ng/mL (**H**). Cells treated with 50 or 100 ng/mL BDNF and 0.1 µM wiskostatin (**I**,**J**). TrkB phosphorylation and SOS1 (**K**) and the integrated density (**L**). HaCaT cells treated with SOS1 (**M**). The effect of nWASP inhibition on TrkB Y816 phosphorylation (**N**). The effect of 0.1 µM wiskostatin and 10 µM 187-1 on TrkB Y816 phosphorylation (**O**). * indicates statistical significance (*p* < 0.05). ns: not significant.

BDNF, as a putative ligand for TrkB and TrkB transcript expression, was examined using PCR in HaCaT cells under 0, 2 and 4 h serum starvation with/without 0.1 µM wiskostatin treatments for 1 h (Figure 7A). Analysis using conventional PCR revealed that the BDNF expression decreased according to the serum starvation in the HaCaT cells (*n* = 2, Figure 7A). The qPCR analysis results were normalised to the 0 h serum starvation (Figure 7B) and to the controls to compare with the 0.1 µM wiskostatin-treated samples (Figure 7C). BDNF was expressed in the HaCaT cells under normal, serum-supplemented culture conditions but following serum withdrawal, the expression of the BDNF transcript was significantly reduced. The treatment with 0.1 µM wiskostatin appeared to have a slight effect on reducing the BDNF transcript expression during normal culture conditions and on increasing the BDNF expression following serum starvation in the qPCR analysis (Figure 7C). The TrkB transcript expression was found to increase following the serum withdrawal—significantly so after 4 h (Figure 7D). The 0.1 µM wiskostatin treatment had no effect on the TrkB transcript under normal culture conditions, but after 2 h, TrkB expression was found to increase when cells were treated with wiskostatin (Figure 7E). Under normal serum conditions, TrkB Y816 phosphorylation was found to be relatively low. The addition of BDNF to HaCaT cells growing at low confluency under these conditions induced TrkB Y816 signalling (Figure 7F). After serum starvation, where TrkB Y816 phosphorylation increased, the addition of BDNF reduced the TrkB Y816 signalling in a similar fashion to the nWASP inhibitor application (Figure 7F). The addition of BDNF between 50 and 150 ng/mL concentrations increased the resistance measured at 4 kHz following cell seeding (Figure 7G,H). The effect of dual treatments with 0.1 µM wiskostatin and 50 ng/mL or 100 ng/mL BDNF was also observed (Figure 7J,K, respectively).

### 3.12. nWASP Inhibitor Treatment May Have Affected the TrkB Signalling through Grb2

In an attempt to explore how nWASP inhibitors may be able to alter the phosphorylation status of TrkB, the effect of SOS1, which is a small-molecule inhibitor that acts to inhibit the activity of Grb2, on TrkB Y816 was examined. The SOS1 treatment at 0.1 and 1 µM concentrations inhibited TrkB Y816 phosphorylation in the HaCaT cells at low confluency and under serum starvation (Figure 7L); the MSOS1 treatments appeared to increase the attachment and spreading of HaCaT cells (Figure 7N).

### 3.13. nWASP Knockdown and TrkB Signalling

The effect of nWASP knockdown on TrkB signalling in HaCaT cells was examined. The cells were serum starved for 4 h and the effect on TrkB Y816 and PLCγ1 Y1253 phosphorylation was then examined using Western blotting (Figure 7O). nWASP knockdown was found to increase TrkB signalling through tyrosine 816 under serum-starved and low confluency conditions. TrkB Y816 activity was examined following prolonged nWASP inhibitor and serum starvation treatment for 4 h. These results demonstrated that the nWASP inhibitor treatment had the same effect of inhibiting TrkB Y816 phosphorylation even when applied from the beginning of the serum withdrawal period (Figure 7).

### 3.14. TrkB Expression in Human Chronic Wound Tissues

**Figure 8:**
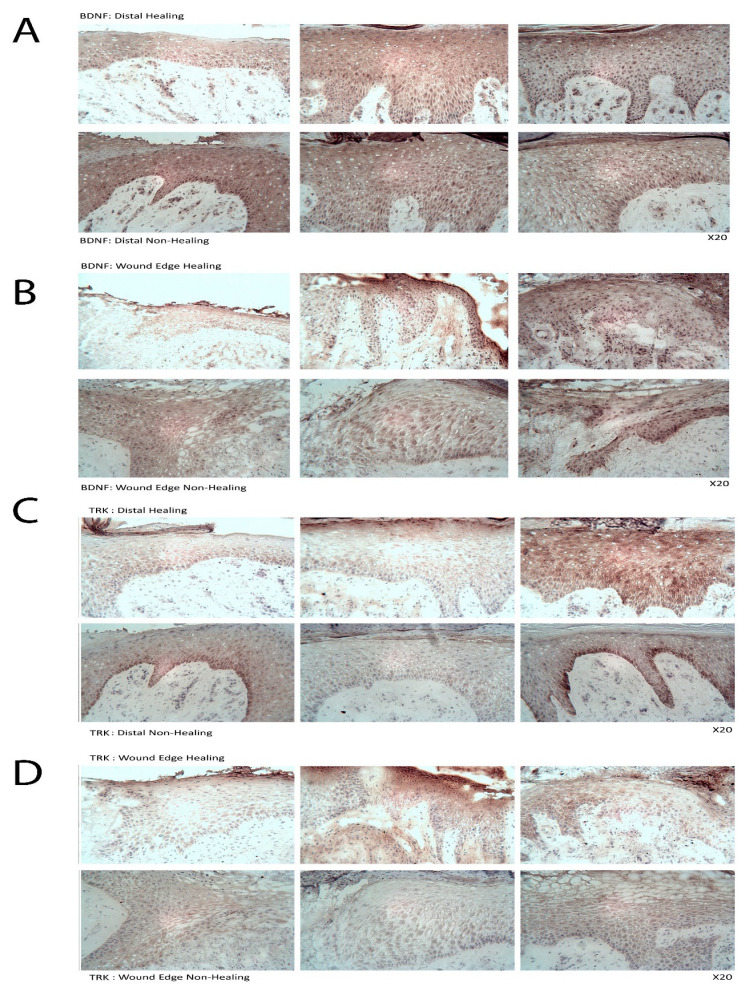
**BDNF and TrkB expression in human chronic wound tissues.** IHC of BDNF protein expression in healing and non-healing chronic wound tissues at distal (**A**) and wound-edge (**B**) locations. IHC of TrkB at distal (**C**) and wound-edge (**D**) locations. Arrows indicate areas of high-intensity staining. Two representative images of each group are shown; ×20 magnification was used.

Following the discovery of a link between nWASP and TrkB and that nWASP is overexpressed in non-healing chronic wounds, the expression of TrkB and BDNF in chronic wounds was examined using IHC on human chronic wound samples. Overall, BDNF showed increased expression in healing (distal) compared with non-healing wounds (Figure 8A,B). TrkB was found to be expressed at higher levels in the basal epidermal cells at sites distal from the wound edge in samples taken from non-healing wound tissues compared with healing tissues (Figure 8C,D).

## 4. Discussion

This study identified nWASP as a molecule that is important in human wound healing and recognises nWASP as a new molecular target to encourage healing in human chronic wounds. Non-healing chronic wounds showed higher expression of nWASP than healing chronic wounds. This quantitative transcript analysis suggests that a balance of nWASP activity in human wounds may be key for healthy wound healing behaviour with over-expression above a certain level indicative of impaired wound healing. Hence, this study proposes that targeting nWASP in a clinical setting may provide a means to encourage healing behaviour in chronic wounds.

In vitro work carried out in this study supported the idea that altering nWASP activity through inhibitor treatment and at a transcript expression level can affect human keratinocyte and endothelial behaviour. The reduction of nWASP activity through nWASP inhibition and transcript expression knockdown affected the cell spreading and attachment properties of cells. Decreased nWASP activity from wild-type keratinocyte levels through transcript knockdown or inhibition appeared to increase the adherent properties of cells; furthermore, the knockdown of nWASP in endothelial cells caused the opposite effect in that attachment and spreading appeared to be impaired. This suggests that nWASP may have a role in the attachment properties of cells to a surface. This could explain how a balance of nWASP expression is important for correct wound healing behaviour, as the optimal level of adhesion and attachment of cells were shown to be a key factor in their migratory properties [9,10]. The implication that nWASP may be involved in affecting these properties of cells may explain how this molecule is involved in wound healing in humans. The molecular mechanisms involved in nWASP activity affecting the attachment properties of keratinocytes in this context are currently unknown but the diverse roles of nWASP and the effects of the actin polymerisation that can result from its activation offer many potential explanations for how nWASP may influence cell attachment and motility.

Using *ex vivo* and in vivo models, this study also demonstrated how nWASP inhibition can encourage wound healing behaviour in tissues with impaired healing abilities and thus provides a new management model for chronic wounds. Using mouse models to study the effect of nWASP inhibitor treatment on wound healing has not only supported previous findings that nWASP has potential as a target in wound healing but highlights a very simple but possibly life-changing therapy option that has potential in human cases. The fact that commercially available nWASP inhibitors can be applied so simply using carrier gels that are already used to treat chronic wounds in the clinic but with such a dramatic effect on encouraging healing in wounds in mice that naturally exhibit impaired wound healing abilities is very promising.

To begin to understand how nWASP inhibitors affect cell behaviour and may act in the context of a chronic wound, an investigation into the effect of altering nWASP activity on the signalling mechanisms in cell models that represent the skin and wound environment was carried out. Initially, a protein array highlighted changes in the expression and phosphorylation of hundreds of kinases and other common signalling proteins following treatment with wiskostatin in HaCaT cells. Of the most significantly altered proteins, several proteins belonging to very common signalling pathways were highlighted, including members of the Ras–Raf–Mek–Erk, Akt–mTOR and Jak–STAT pathways. Numerous receptors and membrane-bound proteins were also identified as being significantly altered by wiskostatin treatment, including VEGFR2 and -3, EGFR and the progesterone receptor, which have been shown to be upstream of these common signalling cascades. TrkB signalling was found to be significantly altered by nWASP inhibitor treatments in HaCaT cells and, as such, the effect of nWASP activity on TrkB signalling became the focus. The findings that were reported here appeared to demonstrate that the TrkB activity in HaCaT cells was extremely sensitive to confluency, serum starvation, BDNF application and nWASP inhibition. Furthermore, the TrkB activity is found to be increased in basal keratinocytes in chronic wound tissues. How TrkB, BDNF and nWASP activity may be linked in the context of chronic wounds requires significant further investigation, especially considering the sensitivity to factors such as confluency and serum availability of the TrkB pathway. The novel relationship between nWASP and TrkB and the downstream PLCγ1 signalling pathway that is affected by changes in signalling may have implications in terms of cell differentiation. As such, further investigation into the factors that affect this signalling and how this may translate into the wound environment needs to be explored.

It is clear that nWASP and TrkB signalling are linked in HaCaT cells and possibly in the chronic wound environment. How nWASP activity alters TrkB signalling and why this pathway is sensitive to factors such as confluency and serum starvation are yet to be answered. The established role of nWASP in endocytosis and receptor trafficking could represent one such potential link. nWASP and TrkB were shown to share numerous interaction partners that may facilitate this activity, for instance, Grb2, pacsin and Nck. A link between nWASP and TrkB through the common binding partner Grb2 has begun to be explored through the use of SOS1. This is an inhibitor of Grb2 activity that acts via blocking the SH3 binding domain of the guanine nucleotide exchange factor SOS, which is the ligand for the adaptor protein Grb2. This blocks the SOS/Grb2 interaction and prevents Ras activation via receptor tyrosine kinases, such as TrkB. SOS1 was found to act in the same way as nWASP inhibition in that TrkB Y816 phosphorylation was significantly reduced at low confluency and after 4 h serum starvation in HaCaT cells. Similarly, Grb2 inhibition was found to increase the resistance of HaCaT cells using ECIS, similar to nWASP inhibitors. This is only the first step in exploring the link between TrkB and nWASP, but this work identified an avenue for further study.

In summary, this study demonstrated that nWASP activity in human wounds can be indicative of its ability to heal effectively and, as a result, using nWASP inhibitors can influence the healing behaviour of wounds. Functional assays indicated that the mechanism through which nWASP may influence wound healing behaviour is potentially via affecting the attachment properties of cells and that this can be mediated through the BDNf/TrkB pathway. This study recognised nWASP as an important therapeutic target in human chronic wounds and proposes the use of nWASP inhibitors as a simple and effective means to encourage wound healing in difficult wounds.

## Figures and Tables

**Table 1:** Significant changes in protein expression and phosphorylation following the wiskostatin treatment.

Protein Name	Phospho Site	%CFC	Protein Name	Phospho Site	%CFC
Decreased			Increased		
PKCI	Pan-specific	−95	RSK1	Pan-specific	248
RSK1/2/3	T573	−92	HSF4	Pan-specific	228
Rb	S795	−91	Fos	T232	227
Trail	Pan-specific	−89	alF2a	S52	225
Tyrosine hydrolase	S40	−89	Mnk2	Pan-specific	239
Progesterone receptor	S294	−85	Integrin α4	S988	325
PTEN	Pan-specific	−81	P70 S6K	T421/S424	337
Smac/DIABLO	Pan-specific	−78	Yes	Pan-specific	327
TYK2	Pan-specific	−81	Akt2 (PkBb)	Pan-specific	386
VEGFR2	Y1214	−74	PTP-PEST	Pan-specific	669
GSK3a	T19+pS21	−71			
SPHK2	Pan-specific	−76			
TTK	Pan-specific	−75			
VGFR3	Pan-specific	−76			
Src	Pan-specific	−74			
Rad17	S645-72	−72			
p38a MAPK	T180/Y182	−65			
Smad2/3	Pan-specific	−67			
MEK6	Pan-specific	−66			
Sik2	Pan-specific	−68			
WNK3	Pan-specific	−67			
Smad1	S465	−59			
ERK2	Pan-specific	−68			
RONa	Pan-specific	−62			
Tau	S422	−62			
ZIPK	Pan-specific	−65			
SHIP2	Pan-specific	−64			
PLC R(PLCγ2)	Pan-specific	−61			
STAT3	Pan-specific	−61			
EGFR	Pan-specific	−63			
SG2NA	Pan-specific	−61			
DUSP3	Pan-specific	−60			
Tau	S199/202	−57			
Jun	S243	−53			
Rb	T821	−57			

## Data Availability

Not applicable.
